# Hepatitis B x (HBx) as a Component of a Functional Cure for Chronic Hepatitis B

**DOI:** 10.3390/biomedicines10092210

**Published:** 2022-09-07

**Authors:** Mark A. Feitelson, Alla Arzumanyan, Ira Spector, Arvin Medhat

**Affiliations:** 1 Room 409 Biolife Building, Department of Biology, College of Science and Technology, Temple University, 1900 N. 12th Street, Philadelphia, PA 19122, USA; 2 SFA Therapeutics, Jenkintown, PA 19046, USA; 3 Department of Molecular Cell Biology, Islamic Azad University Tehran North Branch, Tehran 1975933411, Iran

**Keywords:** functional cure, hepatitis B x antigen, pathogenesis, chronic liver disease

## Abstract

Patients who are carriers of the hepatitis B virus (HBV) are at high risk of chronic liver disease (CLD) which proceeds from hepatitis, to fibrosis, cirrhosis and to hepatocellular carcinoma (HCC). The hepatitis B-encoded X antigen, HBx, promotes virus gene expression and replication, protects infected hepatocytes from immunological destruction, and promotes the development of CLD and HCC. For virus replication, HBx regulates covalently closed circular (ccc) HBV DNA transcription, while for CLD, HBx triggers cellular oxidative stress, in part, by triggering mitochondrial damage that stimulates innate immunity. Constitutive activation of NF-κB by HBx transcriptionally activates pro-inflammatory genes, resulting in hepatocellular destruction, regeneration, and increased integration of the HBx gene into the host genome. NF-κB is also hepatoprotective, which sustains the survival of infected cells. Multiple therapeutic approaches include direct-acting anti-viral compounds and immune-stimulating drugs, but functional cures were not achieved, in part, because none were yet devised to target HBx. In addition, many patients with cirrhosis or HCC have little or no virus replication, but continue to express HBx from integrated templates, suggesting that HBx contributes to the pathogenesis of CLD. Blocking HBx activity will, therefore, impact multiple aspects of the host–virus relationship that are relevant to achieving a functional cure.

## 1. Introduction

HBV is still a major etiologic agent of CLD and HCC despite the availability of an efficacious vaccine and the virtual elimination of HBV from the worldwide blood supply [1]. The reason for this includes a global vaccine coverage of only about 37%; there are still an estimated 260 million carriers and nearly 900,000 HBV-related deaths annually [2]. This unmet medical need was addressed, in part, by the development and clinical application of a variety of nucleoside analogs which suppress virus replication, but these are not curative [3].

HBV is not a lytic virus [4], meaning that liver cell destruction results from the development and persistence of immune responses against virus-infected cells; it is, therefore, generally accepted that the pathogenesis of CLD is immune-mediated. While recent research focused upon the complete eradication of HBV by direct-acting anti-viral drugs, there is also evidence that chronic hepatitis B (CHB) is associated with anti-viral immune responses and autoimmune manifestations, suggesting that stimulation of pro-inflammatory immune responses against the virus may also potentiate auto-reactivity arising from the lysis of virus-infected hepatocytes [5]. Continued development of immune modulating compounds against HBV is, therefore, also important [5].

**Figure 1:**
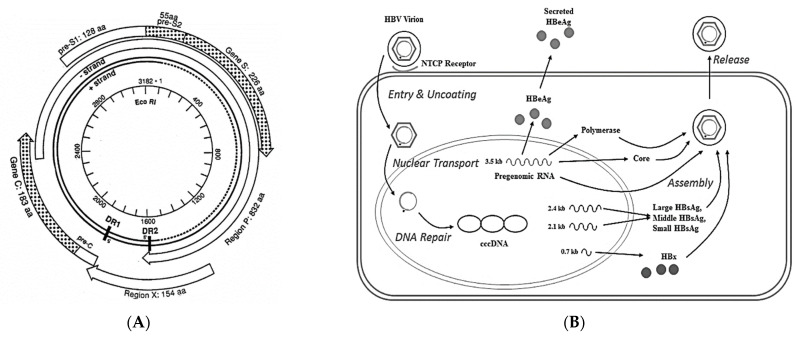
(**A**) Structure of the HBV genome as it exists in the virus particle. The open reading frames encoding the HBsAg polypeptides (gene S and preS), nucleocapsid (gene C), polymerase (region P) and HBx (region X) are shown. The genome has two direct repeat (DR) sequences: DR1 is adjacent to the nick in the long strand of DNA at the end of region X, which is the site on the HBV genome which integrates into host DNA. The short strand is of different lengths in individual virus particles. When the endogenous DNA polymerase inside virus particles fully lengthens this strand (so that the dotted line becomes solid), the DR2 is adjacent to the nick in the short strand [16]. (**B**) When HBV infects a hepatocyte, the partially double-stranded virus DNA becomes fully double-stranded, and then appears in the nuclei of cells as a minichromosome where ccc HBV DNA complexes with host DNA-binding proteins. The minichromosome then acts as a template for the production of all the virus-encoded mRNAs, including the pre-genomic RNA. The latter migrates into the cytoplasm and takes up residence with the virus polymerase in nucleocapsid particles, where it is reverse transcribed into partially double-stranded virus DNA, which then acquires an envelope containing HBsAg polypeptides as it buds from the cell [17]. Abbreviations: ccc—covalently closed circular, DR—direct repeat, HBV—hepatitis B virus, HBsAg—hepatitis B surface antigen, NTCP—sodium taurocholate co-transporting polypeptide (the cell-encoded receptor for HBV).

The HBV genome is a relaxed circular molecule in virus particles that has four open reading frames (ORF) (Figure 1A). The S gene encodes a family of hepatitis B surface-antigen (HBsAg) polypeptides that are on the envelope of the mature virus. The C gene encodes the core protein, which polymerizes into a nucleocapsid having icosahedral symmetry, and which packages the virus genome. The P region encodes the polymerase of the virus. The DNA-dependent DNA polymerase activity converts the partially double-stranded virus genome into a fully double-stranded molecule (by filling in the dotted line in Figure 1A). This relaxed double-stranded molecule, which has a nick at a unique position in each strand adjacent to the direct repeat (DR) sequences, is ligated and appears in the nucleus as supercoiled DNA in the form of a mini-chromosome (Figure 1B). The ccc HBV DNA mini-chromosome is then transcribed into several RNAs, including a pre-genomic RNA, the latter of which is packaged with the virus polymerase into core particles within the cytoplasm. This pre-genomic RNA is then reverse transcribed into minus-strand DNA, followed by RNase degradation of the pre-genomic RNA, and partial plus-strand synthesis prior to budding (Figure 1B).

**Figure 2:**
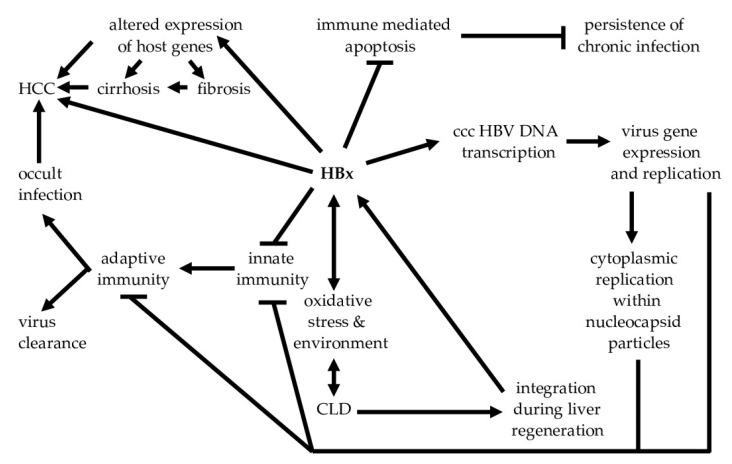
HBx contributes to virus gene expression and replication, which promotes both virus persistence and the pathogenesis of HCC. Details are provided within the text. Abbreviations: HBx—hepatitis B x antigen, CLD—chronic liver disease, HCC—hepatocellular carcinoma.

The fourth ORF, the X region (Figure 1A), encodes the hepatitis B x antigen, HBx, which has pleiotropic properties that support virus gene expression and replication, increase the resistance of infected hepatocytes to immune-mediated lysis, and contribute importantly to the development of HCC [6] (Figure 2). HBx is a *trans*-regulatory protein that impacts virus and host gene expression by binding to other proteins. In the cytoplasm, HBx constitutively activates a number of signal transduction pathways that are pro-inflammatory and pro-carcinogenic [7,8]. In the nucleus, HBx binds to and alters the activity of transcriptional complexes. It also effects the expression and activity of epigenetic regulators of gene expression, such as histone deacetylases and DNA methyltransferases [8]. These properties of HBx have important biological implications. For example, HBx-mediated alterations in the patterns of host-cell gene expression may contribute to CLD by stimulating the expression of pro-inflammatory genes, and the destruction of hepatocytes over time may also trigger auto-reactivity. HBx promotes virus replication by activating virus transcription from ccc HBV DNA in the nuclei of infected cells, and this in turn promotes the persistence of virus antigens that serve as targets for anti-virus specific immune responses. Thus, HBx plays a central role in virus replication and CLD (Figure 2). 

While the clearance of ccc HBV DNA was sought as a goal to permanently eradicate the virus, this is difficult to achieve. This is due to the persistent expression of HBx from ccc HBV DNA and from integrated HBV DNA templates. With each bout of CLD, fragments of the HBV genome become integrated into various sites within the host genome. Prior to becoming a covalently closed circular molecule, HBV DNA exists as a relaxed circle (Figure 1). The X ORF is just upstream from the nick in the long strand of the HBV genome and integrates within replication forks of cellular DNA during liver cell regeneration following a bout of hepatitis. Many of these integrated templates express functional HBx [9] which supports virus gene expression and replication [6] (Figure 2). Thus, a sterilizing cure may be difficult to achieve. Accordingly, investigations have set out to achieve a functional cure, which is characterized by clearance of the HBsAg from blood, reduction of HBV DNA levels in blood to undetectable levels, and resolution of CLD [10]. Since the S gene also becomes integrated into the host chromatin, it is likely that HBsAg will persist in the blood even when ccc HBV DNA is completely eliminated [11]. 

The potential importance of HBx to the pathogenesis of CLD and development of HCC is underscored by the existence of HBx transgenic mice. In these models, persistently high levels of HBx expression are associated with the development of HCC without accompanying virus replication [12,13]. The importance of CLD which progresses from hepatitis to fibrosis, cirrhosis and finally HCC is supported by epidemiological observations that only carriers with CLD are at high risk for HCC, while carriers without liver disease rarely develop HCC [14]. It was subsequently shown that carriers with CLD have integrated templates that make HBx [15]. In this context, the central role of HBx to virus replication, resistance of infected hepatocytes to immune-mediated elimination, and to the development of HCC (Figure 2), suggest that interventions against HBx will bring this infection closer to a functional cure. 

## 2. HBV Treatments and Their Limitations in Achieving a Functional Cure (Table 1)

Persistent virus replication among HBV carriers with CLD increases the risk of HCC, while inhibition of virus replication is often associated with diminished or resolution of CLD [4]. Based on these observations, efforts over the years aimed at the development of anti-viral compounds that target virus replication. In the years following the discovery of HBV, injectable interferon (IFN) (e.g., Intron A from Merck, New York, NY, USA) was developed for treatment, but antiviral efficacy was observed in a minority of patients [18], and the high doses used were accompanied with serious adverse effects, which limited their applicability [19]. Intron A was replaced by pegylated IFN (Pegasis from Genetech, San Francisco, CA, USA) which had the advantage of a longer half-life and milder adverse effects, but a cure was achieved in only 3–11% of patients [19,20]. 

**Table 1:** Therapeutic Approaches Aiming to Cure HBV Infection [20].

Drug *	Mechanism of Action
Intron A, Pegasys	Immunostimulation with interferons
Epivir, Hepsera, Baraclude, Tyzeka, Viread, Vemlidy, Levovir, Besivo	Nucleoside analogs that inhibit the HBV polymerase
VIR-2218, RG6346, JNJ-3989, AB-729	Inhibitory RNAs blocking HBV gene expression
Vebicorvir, Morphothiadin, JNJ 56136379, EDP-514, R07049389, QL-007, ABI-H3733, ZM-H1506R, ALG-000184, B-836, VNRX-9945	Inhibitors that disrupt nucleocapsids or prevent normal nucleocapsid formation
NASVAC, GS-4774, HepTcell, VBI-2601, VVX001, VTP-300, CVI-HBV-002, AIC 649, HB-110, JNJ 64300535, CARG-201	Therapeutic vaccines to target immunological resolution of the carrier state
Selgantolimod, RG7854	Toll-like receptor agonists to stimulate innate immunity
Lenvervimab, Vir-3434	Monoclonal antibodies against HBV antigens
ASX22, GS 4224	PD-L1 inhibitors to overcome T cell exhaustion
Bulevirtide, Hepcludex, hzVSF	Inhibitors of virus entry into susceptible cells

* interferons and nucleoside analogs have been FDA approved and are in the clinic while the other approaches are in human clinical trials.

Nucleoside analogs were then developed as oral medications that targeted the virus polymerase as DNA-chain terminators (Table 1). Nucleoside analogs were efficacious in quickly reducing virus titers in the blood and ameliorating CLD with few adverse effects [19,20]. The first nucleoside analog approved for treatment was lamivudine (Epivir from GSK, Brentford, UK), which reduced both virus titer and ameliorated CLD while under treatment, but resistance developed in up to 30% of treated patients per year [21,22], limiting its effectiveness. Based on the success of lamivudine, many companies pursued similar compounds. Accordingly, adefovir dipivoxil (Hepsera from Gilead, Foster City, CA, USA) was developed to treat patients who became lamivudine resistant, but resistance to adefovir appeared in 20–29% of patients after 5 years of therapy [23,24]. Subsequent compounds in widespread use today include entecavir (Baraclude from BMS, Milan, IL, USA) and tenofovir (Viread from Gilead) [19]. Other direct-acting virus-polymerase inhibitors were also approved for use (Table 1). Although highly effective, virus and disease relapse occurred following the termination of treatment, and none of them was curative [25]. Most patients under treatment remained HBsAg positive, and continuing therapy long term ran the risk of toxicity, resistance, and mounting cost [19]. In addition, there were cross-resistant mutations in the virus polymerase shared by lamivudine and entecavir, so that the probability of developing entecavir-resistant variants was reportedly more than 50% among patients with disease refractory to lamivudine [19]. Combination therapy with pegylated IFN and tenofovir yielded results that were slightly better than either alone, while combinations including tenofovir and entecavir showed no additional virus suppression or disease suppression than either alone [19].

Chronic HBV infection is associated with a weak induction of innate immunity which is mediated through toll-like receptor (TLR) signaling [26]. Appropriate stimulation of innate immunity is important for corresponding adaptive immune responses that clear a virus. Among HBV carriers, induction of innate immunity, and corresponding reduction in HBV levels in the blood were observed with pegylated IFN and entecavir treatment [19]. TLR agonists (Table 1, [20]) reduced HBV DNA levels in the blood of HBV transgenic mice by activation of nuclear factor kappa B (NF-κB) signaling and elevated expression of IFN and other pro-inflammatory cytokines [26], suggesting the viability of this approach. There is increasing evidence that HBV proteins inhibit innate signaling [26]. For example, HBsAg, HBx, and the virus polymerase inhibit signaling pathways that stimulate IFN expression [26,27]. Thus, therapeutic approaches that target HBx activity would likely attenuate the inhibition of innate signaling, thereby promoting immune clearance of HBV. ccc HBV DNA, which is the template for pre-genomic RNA and other virus transcripts, exists in the form of a minichromosome in the nuclei of infected cells (Figure 1B) [17,28]. In addition, HBV replication occurs within cytoplasmic nucleocapsid particles [28,29]. In both cases, virus nucleic acids are sequestered and do not bind to innate immune sensors. HBx promotes ccc HBV DNA transcription via epigenetic modulation of histone acetylation, demethylation, and inhibition of ccc HBV DNA transcriptional repression [30], which is needed for the translation of virus proteins and formation of cytoplasmic replication complexes. Thus, blocking HBx would further permit recovery of innate and adaptive immunity against virus-infected cells, thereby contributing to a functional cure. 

Small-interfering RNAs (siRNAs) that block the expression of one or more HBV genes are under development [31] (Table 1), but it is not clear whether they could be delivered to all infected cells, at sufficient concentrations, and for a sustained period of time, to be therapeutically useful. It is too early to know whether they could augment nucleoside analogs or other therapeutic approaches. There is also the question as to whether siRNAs could block expression of integrated HBV sequences, which often encode HBx and sometimes preS/HBs-containing sequences. However, high levels of HBsAg in the blood are thought to promote immune tolerance, while siRNA inhibition of HBsAg secretion may have both anti-viral effects (reduced HBV DNA in the blood) and attenuation of immunological tolerance [32]. Recently, several siRNAs (e.g., RG6346 from Roche, Basel, Switzerland) reduced the levels of HBsAg in blood, and in some cases, viral titer. The hope is that this approach will be applicable to future combination therapies [2]. 

Small compounds and monoclonal antibodies that block the entry of HBV into susceptible cells provide another potential option [33] (Table 1). For example, Bulevirtide (myrcludex B) is a lipopeptide spanning a portion of the pre-S1 domain of HBsAg. Although it binds the virus receptor on susceptible cells, which results in the reduction in serum HBV DNA, HBsAg levels were not altered [2]. Neutralizing-HBV monoclonal antibodies demonstrated in vivo prophylactic and therapeutic efficacy in animal models, and decreased HBsAg and HBV DNA levels in infected humans [33]. However, following a bout of hepatitis, virus-infected cells undergo regeneration, promoting the intrahepatic spread of the virus without dependence on the production of extracellular virus, suggesting that monoclonal antibodies will have limited utility. 

Gene editing aims to disable (through mutation) or remove HBV sequences by targeting both ccc HBV DNA and integrated virus sequences. Most of the work to date used the CRISPR/Cas9 system, which in multiple studies successfully targeted degradation of ccc HBV DNA [34,35,36]. Hepatotropic viruses, such as adenoviral and adeno-associated viruses, were used as vectors for CRISPR/Cas9, but it is not clear whether this could result in targeting all infected cells. Of course, the expectation is that significant reductions in HBsAg and ccc HBV DNA by gene editing will promote the generation of immune responses that will clear the remaining virus. However, if HBx continues to support virus gene expression and replication, it may still be difficult to achieve a functional cure. Multiple applications of CRISPR/Cas9 could also select for mutations that are resistant to cleavage, especially among integrated virus templates that often encode HBx and HBsAg polypeptides. Off-target cleavage is also a potential limitation, although the use of guide RNAs to conserved-virus sequences simultaneously targeting multiple virus genes may improve the efficacy of this approach [37]. Moreover, double-stranded DNA breaks resulting from gene editing may promote HBV DNA integration, which potentially would increase the risk of hepatocarcinogenesis [38]. 

Over a dozen companies are developing capsid inhibitors [33] (Table 1). Since HBV replication occurs within nucleocapsid particles [29], inhibition of nucleocapsid assembly or disruption of preformed nucleocapsids will interrupt virus replication [33]. This may contribute to the development of potent direct-acting anti-viral combination therapies. In a preclinical mouse model supporting HBV replication, combination therapy using a capsid inhibitor and nucleoside-analog-suppressed virus to a greater extent (>4 logs reduction) than either treatment alone (2.5–3 logs reduction). Virus rebound was observed following the end of treatment [39], suggesting that even combination therapy did not clear ccc HBV DNA. Combination therapy in patients with pegylated IFN also suppressed virus replication more than either monotherapy [40], but HBsAg levels were unaffected, suggesting that even when the pool of ccc HBV DNA is suppressed, the current technology is far from achieving a functional cure. Targeting multiple virus antigens was successful in controlling human immunodeficiency virus infection, and in curing hepatitis C [41]. Unlike hepatitis C, however, portions of the HBV genome become integrated into multiple sites within the host chromatin, and this probably contributes to the ongoing production of HBsAg. In addition, the production of HBx from many of these templates protects cells against immune-mediated clearance, thereby promoting virus persistence and CLD [42] (Figure 1B and Figure 2). 

Many companies are also pursuing therapeutic vaccines designed to target and eliminate virus-infected cells and reduce the risk of CLD progression to cirrhosis and HCC (Table 1). Most of the therapeutic vaccines target HBsAg because clearance of HBsAg during infection is associated with the clearance of virus replication and termination of the carrier state. However, the liver is normally an immunotolerant environment to limit reactivity against gastrointestinal antigens [43]. This immunotolerance may be the reason that HBsAg is not often cleared during natural infection and following therapeutic immunization. Once the carrier state is established, roughly 70% of patients remain asymptomatic, and among those with CLD, very few are capable of clearing the virus from the liver and undergoing seroconversion from HBsAg to anti-HBs. Instead, many patients with CLD develop cellular immune responses which cause liver damage but do not effectively clear the virus-infected cells. With this in mind, several companies are developing checkpoint inhibitors that will permit reactivation of exhausted T cells and subsequent greater antiviral efficacy. However, targeting PD-1, CTLA-4, or other individual receptors with the aim of ameliorating T-cell exhaustion may fall short because there are many other receptors on T cells that could potentially maintain the exhaustion phenotype [44]. 

Virus replication, by itself, does not drive pathogenesis, since HBV is a non-cytopathic virus [4]. Thus, the majority of carriers with very high levels of HBV replication show no evidence of liver damage. Consequently, strong, specific immune responses against multiple virus antigens, and also perhaps host antigens, are likely to drive pathogenesis independent of virus replication. As described below, HBx is likely to be a viable therapeutic target because it supports virus replication, increases the resistance of infected cells to immune elimination, and promotes the development of fibrosis and HCC [42] (Figure 2). Existing and developing therapies target virus-infected cells, but not all virus-infected cells replicate the virus. HBx is critical for driving pathogenesis of CLD and HCC. Thus, targeting virus replication and immune stimulation without also targeting HBx will probably not achieve a functional cure. 

## 3. Role of HBx in the Immunopathogenesis of CLD and HCC

As outlined above, the pathogenesis of acute and chronic HBV infection is immune mediated [4]. For example, the contribution of cell-mediated and cytokine-based immunity to the pathogenesis of acute HBV infection in chimpanzees was demonstrated by the depletion of either CD8^+^ or CD4^+^ T cells during early acute infection, which prevented viral clearance and the onset of liver disease [45]. The contribution of cell-mediated immunity to CLD was shown in HBV transgenic Severe Combined Immunodeficient mice adoptively transferred with splenocytes from immunocompetent syngeneic mice [46]. In this model, adoptive transfer gave rise to chronic hepatitis and partial clearance of virus antigens from sera, as well as virus DNA replicative forms, virus RNA, and virus antigens from the liver [46]. Among chimpanzees acutely infected with HBV, the cytolytic activity of CD8^+^ T cells was suppressed by the production of the anti-inflammatory cytokine, IL-10, resulting in diminished T-cell activity that caused liver damage without virus clearance [47]. Interestingly, acute infection of chimpanzees with HBV and acute infection of woodchucks with a hepatitis B-like virus showed apparent cytokine-mediated virus clearance which occurred prior to the onset of a cell-mediated cytolytic response [48,49]. In these cases, acute hepatitis appeared only after most of the virus was cleared from the liver and serum. However, closer examination of chimpanzees and people revealed the persistence of low level ccc HBV DNA following acute, resolving infection [48,50,51,52], suggesting that HBV could assume an occult infection [53] despite strong, timely immune responses. Neutralizing anti-HBs appear only after the virus is cleared from most hepatocytes [48,54], suggesting that these antibodies protect against re-emergence of the virus after recovery. This outcome calls into question whether the various approaches discussed above, aimed at bolstering anti-virus immunity, will be sufficient to achieve a functional or sterilizing cure. 

The immune-mediated pathogenesis of CLD and HCC is associated with ongoing HBV replication, as indicated by the persistence of hepatitis B e antigen (HBeAg) [55,56], which is a surrogate marker of virus replication. However, CLD provides an oxidative environment that activates HBx, which in turn supports virus replication [57]. HBx alters patterns of host gene expression resulting in the upregulation of neoantigens that trigger additional immune responses during CLD. HBx is also a target for both humoral and cellular immune responses [58,59]. Moreover, HBx transgenic mice are capable of triggering CLD which progresses to HCC in the absence of virus replication [60]. Constitutive high levels of intrahepatic HBx expression were also shown to develop HCC even in the absence of CLD [13]. This suggests that CLD promotes the integration of HBx-encoding HBV DNA (Figure 2), and sustained high levels of HBx could act as a complete carcinogen [61]. Thus, HBx promotes the development of cancer hallmarks [62]. None of these HBx transgenic mice developed HCC on a background of cirrhosis. In natural human infections, HCC often appears on a background of cirrhosis but may also appear in carriers without cirrhosis [63]. Although this suggests that high levels of intrahepatic HBx expression contribute to HCC, viral load, and HBV genotypes, integration events near or within cellular oncogenes or tumor suppressor genes, and mutations in multiple virus genes may also contribute [55]. Thus, while HBsAg and HBeAg may be useful surrogate markers that signal increased risk for HCC, the persistent, elevated expression of intrahepatic HBx should be considered a target for achieving a functional cure.

### 3.1. HBx and Mitochondria

**Figure 3:**
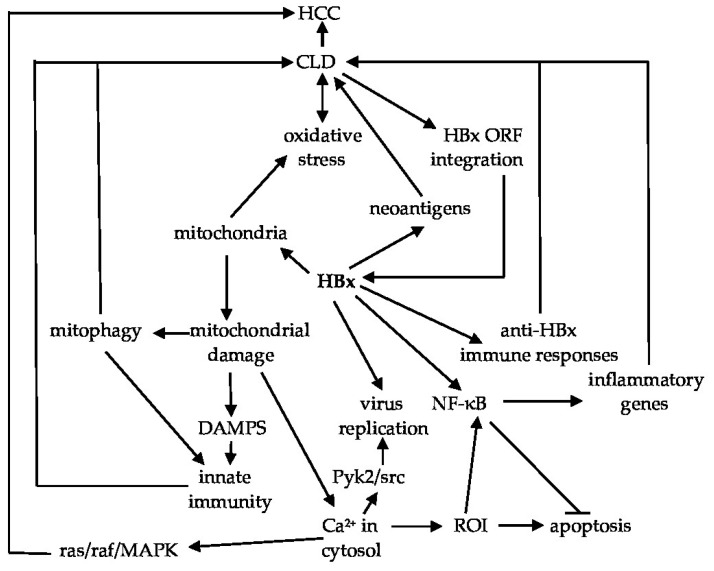
Selected mechanisms whereby HBx contributes to CLD. This includes association of HBx with mitochondria, resulting in mitochondrial damage, mitophagy, and the stimulation of innate immunity through activation of mitochondria-associated MAVS/RIG1. HBx also stimulates virus gene expression and replication [6,74], and promotes systemic inflammation and oxidative stress in the infected cell (which stimulates HBx activity), but at the same time protects hepatocytes from immune-mediated damage via constitutive activation of NF-κB. Hepatocellular regeneration associated with CLD provides opportunities for HBx–ORF integration events, which results in the accumulation of intrahepatic HBx and progression of CLD to HCC. Abbreviations: Ca^2+^—calcium ions, CLD—chronic liver disease, DAMPS—damage-associated molecular patterns, HBx—hepatitis B x antigen, HCC—hepatocellular carcinoma, MAPK—mitogen-activated protein kinase, MAVS—mitochondrial anti-viral signaling protein, NF-κB—nuclear factor kappa B, ORF—open reading frame, Pyk2—proline-rich tyrosine kinase 2, RIG1—retinoic acid-inducible gene I, ROI—reactive oxygen intermediates.

In addition to the oxidative stress triggered by immune responses against virus-infected cells, HBx also stimulates oxidative stress from its association with mitochondria [64] (Figure 3). Oxidative stress damages mitochondrial DNA, and subsequent alterations in cellular bioenergetics cause hepatocellular damage and the release of Damage-Associated Molecular Patterns (DAMPs), which promote inflammation associated with CLD (Figure 2). Cytosolic mitochondrial DNA triggers inflammation via recognition by endosomal-associated TLR-9, and by activation of the inflammasome and IFN pathways [65]. Mitochondria-associated HBx [54] causes depolarization of the mitochondrial membrane potential, promotes the redistribution of calcium ion (Ca^2+^) to the cytosol, and stimulates the generation of reactive oxygen intermediates (ROI), resulting in apoptosis [66,67]. HBx also strongly activates NF-κB [67,68], which is hepatoprotective, in part, by being anti-apoptotic [69]. Thus, many HBx-expressing hepatocytes are protected during bouts of CLD, thereby maintaining cells that support chronic infection. Constitutive activation of NF-κB also results in the upregulated expression of multiple inflammatory genes. The latter promotes a persistent oxidative environment that increases HBx expression and activity, which in turn, stimulates HBV gene expression and replication (Figure 3). HBx activation of phosphoinositol 3-kinase (PI3K) and β-catenin is also hepatoprotective because these block immune mediated cytotoxicity of virus-infected hepatocytes by Fas and by tumor necrosis factor alpha (TNFα) [70]. Elevation of cytosolic Ca^2+^ also (i) induces oxidative stress [71] that subsequently activates Pyk2 and src kinases which support HBV replication, and (ii) elevates Ras, Raf and Mitogen-Activated Protein Kinase (MAPK) signaling, which contribute to cell growth and hepatocarcinogenesis [57]. HBx-elevated ROI activate pro-inflammatory signaling through NF-κB, and some of the pro-inflammatory cytokines activate STAT3, which then transcriptionally regulates cell growth and differentiation [57]. In addition, mitochondria-associated HBx binds to Mitochondrial Antiviral Signaling Protein (MAVS), which is a part of the Retinoic acid-Inducible Gene 1 (RIG-1) pathway that activates type 1 IFN and the expression of other pro-inflammatory genes mediating innate immunity [72]. In summary, HBx within mitochondria enhances HBV replication, helps to confer resistance of infected cells to apoptosis through hepatoprotection despite activating inflammation, and blunts innate immune responses required for mediating virus clearance. Mitochondria-associated HBx also triggers mitophagy [73]. Mitophagy could also promote chronic inflammation, continued virus replication, and progression to HCC (Figure 3). These features suggest that blocking HBx activity will impact multiple aspects of the host–virus relationship that are relevant to achieving a functional cure.

### 3.2. HBx and Fibrogenesis

HBx also contributes to the pathogenesis of CLD by promoting fibrogenesis, which is a well-established risk factor for HCC [75] (Figure 1). The likelihood of developing fibrogenesis correlates with the extent of HBV integration since many integrated templates encode HBx. HBx activates liver stellate cells in vitro to produce Transforming Growth Factor β (TGFβ) which is pro-fibrogenic and pro-carcinogenic [62] via differential phosphorylation of the TGFβ-signaling molecule smad3 [76]. Hepatic stellate cells treated with conditioned medium from HBx-positive hepatocytes showed increased expression of TGFβ and other markers associated with fibrogenesis, such as collagen 1, alpha-smooth muscle actin (αSMA), connective-tissue growth factor (CTGF), and matrix metalloproteinase-2 (MMP-2) [77,78]. This paracrine effect also triggered stellate cell proliferation [77,78]. Further work showed that HBx potentiates TGFβ signaling [79]. HBx suppresses the expression of the natural TGFβ inhibitor, alpha-2 macroglobulin (α2M), by (i) activation of NF-κB, which in turn blocks the expression of the α2M gene normally activated by STAT3, and/or (ii) by the HBx activation of PI3K, which then suppresses α2M expression [70]. Activation of TGFβ signaling and additional markers associated with fibrosis was seen by proteomics analysis of HBx transgenic mice that developed mild fibrosis [80]. For example, HBx transcriptionally promotes the expression of fibronectin via activation of NF-κB and via inhibition of p53, the latter normally suppressing the fibronectin promoter [70,81]. HBx may also contribute to extracellular matrix (ECM) remodeling by stimulating PI3K signaling that elevates expression of the adhesion protein, LIM, and SH3 protein 1 (LASP-1), resulting in increased cellular proliferation and migration [82]. HBx also interacts with Hypoxia Inducible Factor 1α (HIF-1α) which contributes to liver fibrosis by promoting the rearrangement of the normal ECM [83]. In addition, elevated levels of HBx and HIF-1α activate Notch signaling [84,85] which is associated with the development of fibrosis in many organs, including the liver [86]. Interestingly, HBx activation of the *Yes* oncogene-associated protein, YAP (in HIPPO signaling), also contributes to fibrosis by activation of Notch signaling via upregulated expression of the Notch ligand, Jagged 1 [87]. Independent work showed that HBx upregulated the expression of Jagged in HCC [88]. Given that HBx promotes the development of fibrosis by multiple mechanisms, therapeutic intervention aimed at HBx would likely change the pathogenesis of chronic infection which would contribute centrally to the resolution of CLD and a functional cure.

## 4. HBx and cccHBV DNA as Targets for a Functional Cure

A fundamental function of HBx is to support virus gene expression and replication [6,74]. HBx protein was present in the serum of patients replicating HBV [89]. In longitudinal studies, clearance of the virus was accompanied by the loss of HBx in the blood and the appearance of anti-HBx [90]. These events closely followed seroconversion of HBeAg to anti-HBe [90]. Other studies showed increased frequency of both HBx in liver and anti-HBx in serum among patients with liver cirrhosis and HCC compared with patients with hepatitis [58,91]. Independent work showed that HBx *trans*-activated the *X* (*HBx)* and *C* (core) gene promoters both in vivo and in vitro [57,92,93,94], thereby supporting HBV replication. Mutational abrogation of woodchuck hepatitis x antigen (WHx) in the related woodchuck hepatitis virus (WHV) model failed to develop the carrier state and CLD upon experimental infection [95,96], showing that the X antigen was essential for supporting WHV replication in the establishment and persistence of the carrier state. HBx was also shown to be central to supporting HBV replication in cell culture [93] and in HBV transgenic mice [92]. Naturally occurring HBx-deletion variants in human serum samples were also found in patients with mild hepatitis who were HBsAg negative [97], again suggesting that HBx supported HBV replication. HBx-specific siRNAs inhibited virus replication in vitro and in vivo [98]. HBx-specific T-cell epitopes were also identified in vitro and in vivo [99,100], but their role in virus replication and the pathogenesis of CLD are still open questions. 

More recently, the role of HBx in virus replication was clarified. ccc HBV DNA exists as a minichromosome in the nuclei of infected cells [101]. During virus replication, HBx is also localized in the nucleus where it is associated with transcriptional complexes supporting expression of ccc HBV DNA and host genes that mediate hallmarks of cancer (e.g., activation of signaling pathways mediated by ras, src, myc, PI3K, NF-κB, and histone deacetylases) [62,102]. As mentioned above, HBx contributes to virus replication, in part by elevating the levels of Ca^2+^ to activate Pyk2/Src and Focal Activated Kinase (FAK) pathways [103]. HBx-mediated Ca^2+^ signaling also facilitates viral core assembly and pre-genomic RNA (pgRNA) production [103], both of which are required for virus replication (Figure 1). It is proposed that when HBx expression is high enough in infected cells, it results in the stimulation of cell growth. However, among differentiated hepatocytes, the more HBx stimulates cell growth, the more likely the cellular response will be G1 arrest or apoptosis. The latter was shown in HBx transgenic mice after partial hepatectomy [104,105], suggesting that the outcome of HBx expression in hepatocytes, in part, depends upon the state of cellular differentiation. In this context, quiescent hepatocytes optimally support virus replication, so there appears to be a balance between the levels and intracellular localization of HBx and its biological effects upon infected cells and virus replication. In cell nuclei, HBx is associated with the HBV minichromosome, where it epigenetically modulates ccc HBV DNA transcription, in part, by inhibiting the activities of several histone deacetylases [106]. HBx also binds to and inactivates Protein Arginine Methyltransferase 1 (PRMT1) [107] and facilitates the degradation of the methyltransferase WD Repeat Domain 77 protein (WDR77), both of which negatively regulate HBV replication [108]. HBx also facilities the proteasomal degradation of the Structural Maintenance of Chromosomes (SMC) complex, SMC5/6, which attenuates the transcription of ccc HBV DNA [109]. Targeting HBx expression and function in regulating ccc HBV DNA transcription will, therefore, be an important component in achieving a functional cure. 

## 5. HBx Integration

Viral integration appears to be linked to immune-mediated CLD, characterized by the destruction of virus-infected cells and regeneration, which is accompanied by oxidative stress and damage to hepatocellular DNA (Figure 1). Oxidative stress gives rise to double-stranded DNA breaks, which provides opportunities for increased HBV DNA integration [110,111]. The most commonly integrated viral sequences are at the end of the HBV genome, which encodes HBx, and sometimes also includes upstream HBsAg and preS sequences [112]. The more severe the CLD, the more integration events occur throughout the host genome [113], resulting over time in the cytoplasmic accumulation of HBx [114,115,116]. HBx alters multiple-signal-transduction pathways in the cytoplasm (e.g., NF-κB, [117]) that promote resistance of hepatocytes to immunological damage, persistent survival and growth, resistance to apoptosis, and contribute to the appearance of other hallmarks of cancer [62]. This provides positive feedback that amplifies HBx expression and function and helps to explain observations that progressive CLD among HBV carriers is a major risk factor for the development of HCC [14]. Thus, the stimulation of HBx by oxidative stress during CLD suggests that antioxidants could have therapeutic value [118]. This hypothesis was supported by results in HBx transgenic mice that developed CLD and HCC treated with short chain fatty acids (SCFAs) [12]. SCFAs are known to reduce oxidative stress by epigenetically shifting Th1 (pro-inflammatory) to Th2 (anti-inflammatory) cytokines and by promoting the differentiation of naïve T cells into the T regulatory cells [119]. SCFAs downregulate the expression of pro-inflammatory cytokines, such as IFN, interleukin-17 and TNFα, by blocking NF-κB signaling, while upregulating the expression of anti-inflammatory cytokines, such as interleukin-10. SCFAs mediate these changes by binding to cognate receptors and stimulating signaling pathways that modify cellular transcriptional patterns, suggesting that SCFAs may have a therapeutic application in delaying or preventing the pathogenesis of CLD and development of HCC [12]. Independent observations also suggested that the attenuation of T-cell-mediated CLD will reduce the risk of CLD progression to HCC [4]. Targeting HBx with SCFAs reduced HBx expression and activity by attenuating inflammation and oxidative stress [120]. This would be expected to have an anti-viral effect and also reduce HBx activity to epigenetically alter the patterns of host gene expression, thereby permitting restoration of a more normal cellular phenotype [120,121]. This correlates with the decreased frequency of dysplasia and HCC in SCFA-treated HBx transgenic mice compared with those treated with placebo [12]. In this context, even the anti-inflammatory properties of low-dose aspirin significantly reduced the risk of liver disease-related mortality and the development of HCC [122], suggesting that the attenuation of CLD is an important element of a functional cure. 

Integrated virus templates do not produce replication competent transcripts, but do make proteins that promote T-cell exhaustion and HCC [111]. HBx made from the transcription of integrated virus DNA [111] promotes cell survival and growth, thereby permitting the continued synthesis of HBsAg, even among patients who have low levels of virus replication [123]. High concentrations of HBsAg in the blood, whether HBsAg mRNA is encoded by ccc HBV DNA or from integrated templates, may contribute to high zone T-cell tolerance and virus persistence. PreS/S polypeptides encoded from integrated templates accumulate in the endoplasmic reticulum (ER), resulting in ER stress and elevation of ROI, which further promotes DNA damage. The latter provides additional opportunities for HBV DNA integration and continued HBx expression during CLD [113], which further protects infected cells, thereby promoting production of HBsAg [57,93]. These findings suggest that targeting HBsAg will have limited effectiveness because more is always being made from integrated templates containing the HBs gene. If so, then targeting HBx and lowering oxidative stress will also contribute to the reduced expression and clearance of HBsAg made from integrated templates, thereby contributing to a functional cure. Targeting HBx expression/activity will help to re-establish homeostasis among hepatocytes and within the immune system, thereby reducing the risk for the appearance of fibrosis, cirrhosis, and HCC.

HBV integration is also associated with the clonal expansion of hepatocytes in the infected liver, even between bouts of CLD [15,124]. While cis-acting mechanisms related to HBx integration probably contribute to enhanced survival and growth among tumor nodules in some patients, the *trans*-activation function of HBx from many of these integrated templates may be more common. Either way, the risk of developing HCC remains elevated in HBsAg-negative HBV patients and those with occult infections [125,126] (Figure 1), again suggesting that HBx drives pathogenesis even in the absence of HBV replication. This indicates that even when the markers associated with a functional cure (HBsAg and HBV DNA in the blood) are not detectable, a functional cure is not established and CLD may still progress to HCC. 

## 6. Conclusions

The utilization of HBsAg and ccc HBV DNA as markers for a functional cure is based on observations that acute, resolving hepatitis B results in the clearance of these markers and that a small percentage of patients who clear HBsAg also have a reduced risk of the progression of CLD to cirrhosis and HCC [10]. The fact that HBx supports virus gene expression and replication [6,74] suggests that inhibition of HBx will contribute to decreased levels of ccc HBV DNA, thereby contributing to a functional cure. Since (i) HBx activity is promoted in CLD by oxidative stress [64], (ii) integration and intrahepatic accumulation of HBx directly correlate with the progression of CLD [114,115], and (iii) HBx constitutively activates pro-inflammatory molecules, such as NF-κB [67,68], it can be inferred that amelioration of CLD will also attenuate HBx expression and activity, thereby contributing to a functional cure.

Functional cures were achieved in 20–80% of patients with low baseline HBsAg and HBV DNA titers in the blood, who were treated with a combination therapy consisting of pegylated IFN and a nucleoside analog [127]. In these cases, significant reductions in virus titers may help restore innate and virus-specific immune responses in chronic hepatitis B patients [128]. However, patients with high levels of HBV DNA in the blood, who are mostly HBeAg positive, do not achieve a functional cure at high rates when similarly treated, and may relapse after treatment is terminated [127]. Capsid inhibitors, siRNAs, CRISPR/Cas9, and other approaches mentioned above, are still under development and whether singly, or in combination, any of these will contribute to a functional cure remains to be demonstrated. However, none of these approaches take into consideration HBx and its potential contribution to a functional cure. Moreover, none of the clinical trials, completed or underway, specifically targeted HBx, and the point of this review is to suggest that this be done.

In acute, resolving hepatitis B, where little integration occurs, and strong, multi-specific, anti-viral immune responses are present, elements for virus clearance are operative. If CLD is mild, not much integration will occur, which may favor seroconversion in a small proportion of patients. In both cases, hepatocellular destruction and turnover are limited, providing fewer opportunities for integration and HBx expression to occur and persist. However, when CLD progresses to fibrosis and cirrhosis, elevated levels of intrahepatic HBx accumulate [113], which promotes the persistence of virus replication, protects cells from immune-mediated elimination, and mediates the appearance of hallmarks of cancer [61,62]. This may contribute to why it is difficult to achieve a functional cure [129]. HBx also impacts upon protein ubiquitination, proteasome-mediated degradation [130], and apoptosis and anti-viral responses. In addition, many of the properties of HBx are epigenetically mediated [131]. This includes the ability of HBx to recruit and upregulate selected host histone acetyltransferases (which promote activation of ccc HBV DNA transcription) and DNA methyltransferases (which methylate host DNA and contribute to hepatocarcinogenesis) [38], suggesting that targeting epigenetic functions of HBx by SCFAs [12] (or other compounds) could have therapeutic potential. Thus, therapeutic inhibition of HBx expression and function, in combination with one or more of the approaches above, should contribute to a functional cure prior to the development of cirrhosis and HCC.

## Data Availability

Not applicable.

## Abbreviations

α2M	alpha 2 macroglobulin
αSMA	alpha-smooth muscle actin
Ca^2+^	calcium ions
ccc HBV DNA	covalently closed circular hepatitis B virus DNA
CLD	chronic liver disease
CHB	chronic hepatitis B
CTGF	connective-tissue growth factor
DAMPs	damage-associated molecular patterns
DR	direct repeats
ECM	extracellular matrix
ER	endoplasmic reticulum
FAK	focal activated kinase
HBeAg	hepatitis B e antigen
HBsAg	hepatitis B surface antigen
HBV	hepatitis B virus
HBx	hepatitis B x antigen
HCC	hepatocellular carcinoma
HIF-1α	hypoxia inducible factor 1α
IFN	interferon
LASP-1	LIM and SH3 domain protein 1
MAPK	mitogen-activated protein kinase
MAVS	mitochondrial antiviral signaling protein, matrix metalloproteinase-2
NF-κB	nuclear factor kappa B
NTCP	Sodium taurocholate co-transporting polypeptide
ORF	open reading frame
pgRNA	pre-genomic RNA
PI3K	phosphoinositol 3-kinase
PRMT1	protein arginine methyltransferase
Pyk2	proline-rich tyrosine kinase 2
RIG-1	retinoic acid-inducible gene 1
ROI	reactive oxygen intermediates
SCFAs	short-chain fatty acids
Smc	structural maintenance of chromosomes
TGFβ	transforming growth factor β
TLR	toll-like receptor
TNFα	tumor necrosis factor alpha
siRNAs	small interfering RNAs
WDR77	WD repeat domain 77 protein
WHV	woodchuck hepatitis virus
